# The effect of rapid privatisation on mortality in mono-industrial towns in post-Soviet Russia: a retrospective cohort study

**DOI:** 10.1016/S2468-2667(17)30072-5

**Published:** 2017-04-11

**Authors:** Aytalina Azarova, Darja Irdam, Alexi Gugushvili, Mihaly Fazekas, Gábor Scheiring, Pia Horvat, Denes Stefler, Irina Kolesnikova, Vladimir Popov, Ivan Szelenyi, David Stuckler, Michael Marmot, Michael Murphy, Martin McKee, Martin Bobak, Lawrence King

**Affiliations:** a Department of Sociology, University of Cambridge, Cambridge, UK; b Department of Social Policy and Intervention, University of Oxford, Oxford, UK; c Faculty of Politics, Psychology, Sociology and International Studies, University of Oxford, Oxford, UK; d Department of Epidemiology and Public Health, University College London, London, UK; e Institute of Economics of the National Academy of Sciences of Belarus, Minsk, Belarus; f Central Economics and Mathematics Institute, Russian Academy of Sciences, Moscow, Russia; g Sociology Department, Yale University, New Haven, CT, USA; h Department of Social Policy, London School of Economics, London, UK; i Department of Health Services Research and Policy, London School of Hygiene & Tropical Medicine, London, UK

## Abstract

**Background:**

Population-level data suggest that economic disruptions in the early 1990s increased working-age male mortality in post-Soviet countries. This study uses individual-level data, using an indirect estimation method, to test the hypothesis that fast privatisation increased mortality in Russia.

**Methods:**

In this retrospective cohort study, we surveyed surviving relatives of individuals who lived through the post-communist transition to retrieve demographic and socioeconomic characteristics of their parents, siblings, and male partners. The survey was done within the framework of the European Research Council (ERC) project PrivMort (The Impact of Privatization on the Mortality Crisis in Eastern Europe). We surveyed relatives in 20 mono-industrial towns in the European part of Russia (ie, the landmass to the west of the Urals). We compared ten fast-privatised and ten slow-privatised towns selected using propensity score matching. In the selected towns, population surveys were done in which respondents provided information about vital status, sociodemographic and socioeconomic characteristics and health-related behaviours of their parents, two eldest siblings (if eligible), and first husbands or long-term partners. We calculated indirect age-standardised mortality rates in fast and slow privatised towns and then, in multivariate analyses, calculated Poisson proportional incidence rate ratios to estimate the effect of rapid privatisation on all-cause mortality risk.

**Findings:**

Between November, 2014, and March, 2015, 21 494 households were identified in 20 towns. Overall, 13 932 valid interviews were done (with information collected for 38 339 relatives [21 634 men and 16 705 women]). Fast privatisation was strongly associated with higher working-age male mortality rates both between 1992 and 1998 (age-standardised mortality ratio in men aged 20–69 years in fast *vs* slow privatised towns: 1·13, SMR 0·83, 95% CI 0·77–0·88 *vs* 0·73, 0·69–0·77, respectively) and from 1999 to 2006 (1·15, 0·91, 0·86–0·97 *vs* 0·79, 0·75–0·84). After adjusting for age, marital status, material deprivation history, smoking, drinking and socioeconomic status, working-age men in fast-privatised towns experienced 13% higher mortality than in slow-privatised towns (95% CI 1–26).

**Interpretation:**

The rapid pace of privatisation was a significant factor in the marked increase in working-age male mortality in post-Soviet Russia. By providing compelling evidence in support of the health benefits of a slower pace of privatisation, this study can assist policy makers in making informed decisions about the speed and scope of government interventions.

**Funding:**

The European Research Council.

## Introduction

The period from 1990 to 1995 in the newly independent post-Soviet states was marked by an estimated extra 7 million premature deaths, 4 million in Russia alone.^1^ Many of these deaths were due to external causes and cardiovascular diseases.^2^ The group most affected by this rapidly increasing mortality was working-age men.^3^ There is extensive work documenting the importance of alcohol, unstable employment, and stress^4^, ^5^, ^6^, ^7^ as proximal determinants. However, the upstream determinants are less well understood, yet crucially important to inform policy makers in future periods of rapid political and economic transition.^8^ One of the central pillars of the post-communist transition has been privatisation.^9^

Stuckler and colleagues^10^ reported a cross-national association between extremely fast and extensive privatisation (so-called mass privatisation) with higher working-age male mortality, suggesting that unemployment was a primary mechanism linking privatisation and premature deaths. While it is well known that state enterprises hoarded an excessive and often ineffective labour force during the Soviet era, resisting massive layoffs during the post-communist crisis probably indirectly affected public health. Being employed, at least part-time, or even nominally, provided people with minimal security and gave them the feeling of being in control of their lives. Unemployment and related stress led to a hitherto unseen drop in life expectancy.^11^

**Research in context**

**Evidence before this study**

We undertook an extensive analysis of the scientific literature on the effect of economic reforms on public health, morbidity, and mortality in post-communist Russia, and relevant electronic databases. We searched PubMed and Embase for reports published between 1990 and 2013, published in any language, with the following search terms: “mortality in Russia”, “working age mortality”, “male mortality”, “mortality and transition”, “privatization”, “alcohol”. We also searched our own extensive library of literature on mortality in the former communist countries of Europe. These data were highly heterogeneous and had limited utility as they were not suitable for linking the speed of privatisation in various settlements to individuals' mortality. Meta-analysis of the effects of privatisation on the health of employees and populations does not permit drawing a pooled estimate, as most of the estimates apply to countries outside our focus. Evidence from post-communist countries on health outcomes of politico-economic transitions remains inconclusive, although available data suggest that adverse health outcomes could result from rising unemployment and stress levels associated with mass privatisation.

**Added value of this study**

Employing an innovative design, we were able to quantify and compare inequalities in all-cause mortality, caused by policy intervention, during the 1990s in the urban population of Russian mono-industrial towns. We clarify the uncertainty associated with the link between the pace of privatisation in the former Soviet Union and the increasing mortality. This study is unique in using individual-level data collected specifically to study the health effects of privatisation and thus fills gaps in the scientific literature. Methodological contribution includes the use of the propensity score matching method to isolate the effect of privatisation by closely matching the settlements for a prospective survey prior to collecting the data via survey. Indirect demographic techniques were used to show how mortality levels in Russia changed over time. We used the method of establishing a convenience cohort study, based on the Brass indirect method that surveys random population samples to collect data on deaths of respondents' relatives, so as to estimate key population mortality parameters. Most studies focusing on this period examined macro data, while high-quality data on the level of individuals remain scarce. Even though the design of the study was dictated by the need to test the privatisation thesis, a large new empirical base provides rich data for individual-level health risk analysis.

**Implications of all the available evidence**

Our findings contribute to an emerging specialty at the intersection of comparative political economy and public health, and provide an evidence base for the public and scholarly debate on the effect of privatisation on public health. Our multi-level work contributes to the methodological literature, offering a concrete example of how to study the effects of structural socioeconomic changes and individual-level factors on population health. This new research tradition extends the social determinants of morbidity and mortality research programme by linking it to specific political and economic policies and processes. Findings are also relevant for policy makers considering similar interventions in post-communist societies and beyond.

Another possible explanation for the effects of privatisation on health could be the fact that before reforms, large state enterprises offered a wide range of social benefits to employees and their families. This was especially the case for city-forming enterprises that bore substantial responsibilities for the day-to-day wellbeing of their employees by providing company housing, health care, catering, day care, and holiday recreation.^12^, ^13^, ^14^ When these enterprises were privatised, such provisions ceased. Usually, in transition economies these responsibilities were taken over by municipal and local administrative divisions, but due to serious budget deficits, these organisations were unable to provide adequate quality and scope of social services.^15^ The loss of health services can affect directly population health. However, the loss of social services, as well as the uncertainty created by privatisation, could increase levels of stress and associated risky behaviours like excessive drinking, which is a well documented cause of increased post-communist male mortality.^16^ It is estimated that as much as 30% of overall male mortality in Russia is accounted for by excessive drinking, and even more in working ages.^4^, ^17^ King and colleagues^18^ recorded a cross-national association between mass privatisation, reduced health-care resources, unemployment and stress-related outcomes like alcohol consumption, suicides, ischaemic heart disease, and homicide.

Finally, extremely fast and extensive privatisation could be linked to poor health outcomes by damaging firm performance and state capacity. Findings of cross-national time-series analysis, cross-sectional firm-level regressions, and case studies^15^, ^19^ show that at the micro-level, mass privatisated firms experienced reduced innovation, employed fewer employees, lowered economic output, and paid fewer taxes. At the macro-level, mass privatisation was associated with substantially lower economic growth and poorer state performance.^20^, ^21^

While national-level studies can plausibly test macro-mechanisms, critics have noted potential limitations such as omitted variable bias and ecological fallacy (ie, when the factors associated with group-level mortality rates fail to be associated with individual-level mortality).^22^ The aim of this study was to assess the effect of rapid privatisation at the individual level with data collected via survey within the framework of the European Research Council (ERC) Advanced Grant project, The Impact of Privatization on the Mortality Crisis in Eastern Europe (PrivMort).^23^

## Methods

### Study design

The PrivMort project^23^ was designed to retrieve demographic and socioeconomic characteristics of individuals indirectly via surviving relatives. The method, developed by William Brass, was originally designed to estimate rates of vital events (births and deaths in countries with low literacy and numeracy). Because the information about relatives was not collected directly, it is often referred to as indirect estimation or the Brass technique after its developer. This information provides estimates of mortality rates that are acceptably representative of the underlying population.^24^, ^25^ This approach is recommended by the UN for inclusion in censuses in such situations in which alternative official sources are not available^26^ and has been used successfully for analysing adult mortality differentials in Russia.^27^ There were no changes made to the previously published protocol.^23^ The full protocol describes the data collected in all towns that participated in the survey, while this study analyses the data from a smaller survey sample of towns. The remainder are five multi-industrial towns matched with five mono-industrial towns. They are not well matched with the other towns and were selected to test a different set of hypotheses that will be reported elsewhere. These are therefore not studied in this article.

### Study populations

Here we report analysis of data from 20 Russian towns whose economy is dominated by a single company. We selected mono-towns or towns of city-forming enterprise (monogoroda) because such settlements allow us to better isolate the effects of privatisation. Whereas some city-forming enterprises were fully privatised within 1 or 2 years, in others more gradual privatisation strategies were adopted.

Following convention, we defined mono-industrial towns as settlements in which a single major enterprise employed more than 7·5% of total population, and the second largest enterprise had to be at least three times smaller in terms of its share in total employment in 1991. Every town had a population of between 5000 and 100 000 in 1991. An extensive review of sources was undertaken to compile enterprise-level data.^23^ We then classified mono-towns into three groups: towns where the major enterprise had been privatised rapidly, transferring 90% or more of its shares in any 2 consecutive years between 1992 and 1998; towns that adopted a more gradual approach in which less than 50% of shares were privatised in 2 consecutive years between 1992 and 1998, a period that provides sufficient time to account for any lagged effects of privatisation on mortality; and towns with a medium rate of privatisation (defined by privatisation of state shares of between 50% and 90%). We excluded towns with medium-pace privatisation to maximise the contrast in the speed of privatisation between the groups of firms. Future analysis using official gross mortality data and a larger sample of towns will allow us to perform an ecological analysis to assess whether there is a dose–response relationship between privatisation and mortality.

We chose a set of 20 mono-towns in west Russia, closely matched by initial conditions, so that ten towns that experienced fast privatisation (treatment group) were matched to ten that experienced gradual privatisation (control group). Interviews were performed between November, 2014, and March, 2015, by the Russian Agency for Public Opinion Research (VCIOM). The appendix lists the matched mono-industrial towns (appendix p 3). We used standard propensity score matching, logistic regression with no replacement, using the nearest neighbour approach. Every town in the treatment group was matched to a town in the control group with the closest score, so that a unit was selected only once, identifying the top ten pairs with closest propensity scores. We estimated conditional probabilities based on eight potential predictors of mortality, all measured for the pre-transition year, 1991, with the exception of wages, which were not available before 1992. We also used other matching algorithms such as trying alternative measures of the covariates (eg, number of hospital beds rather than number of physicians) and different distance measures (eg, Mahalanobis). The alternative methods explored produced town lists only marginally differing (ie, 1–2 towns changing), leading to the conclusion that the selected matching method is robust. The appendix shows these eight covariates across the matched towns (appendix p 4).

### Study participants

In every settlement, 20 to 45 starting points were identified using a grid. Interviewers were instructed to launch the random walk procedure at a randomly assigned address within each cell selected, inviting face-to-face interviews at every fourth household. The respondents were asked to provide information about vital status, sociodemographic and socioeconomic characteristics and health-related behaviours of their parents, two eldest siblings (if eligible), and first husbands or long-term partners. Ethics approval was obtained from the University of Cambridge Department of Sociology ethics committee and ERC ethics advisers. The data were anonymised to prevent any potential identification of individual respondents.

### Statistical analysis

We calculated indirect age-standardised mortality rates (SMR) in rapidly and slowly privatised towns and then, in multivariate analyses, calculated Poisson proportional incidence rate ratios for men and women separately, with corresponding 95% confidence intervals (CIs) and robust standard errors that control for heteroscedasticity, to estimate associations of relatives' characteristics and speed of privatisation with the risk of death from all causes. Regression models were estimated with Stata (version 12.0).

The multivariate analysis focuses on the effect of the speed of privatisation on mortality of those of working age, who experienced the greatest increase in mortality during the post-communist transition.^28^ Relatives younger than 20 years or older than 69 years in 1992, those who died before the beginning of the reforms (in 1991 or earlier) or did not have full information on education, marital status, professional occupation, material deprivation, alcohol drinking and smoking habits were excluded from the analysis. We also excluded those who were not residing in the 20 matched towns during privatisation, that is, for most of the 1990s. Work-related data were collected only on relatives who had not retired by 1992.

### Role of the funding source

The funder had no role in study design, data collection, analyses, and reporting. AA, DI, LK, MMu, AG, GS, MB, DSte, and PH had access to raw data. The corresponding author had full access to all of the data and the final responsibility to submit for publication.

## Results

Between November, 2014, and March, 2015, 21 494 households were identified in 20 towns. In 647 households, nobody could be contacted (after five visits), a further 5473 declined to be interviewed, and 732 were unable to participate due to physical or mental impairment. Overall, 14 642 interviews were completed. After data cleaning, a sample was reduced to 13 932 valid interviews, with a 65% response rate. Information about 38 339 relatives (21 634 men and 16 705 women) was collected. After exclusion criteria were applied, 19 167 were eligible for inclusion (12 086 men and 7081 women). Table 1 presents the availability of data for individuals in towns of each type and by sex. The distribution of people by vital status, education, occupation, and other characteristics in both types of town was similar.

**Table 1:** Independent variables for fast and slow privatised towns

		**Fast-privatised towns**		**Slow-privatised towns**	
		Men	Women	Men	Women
People		5018 (62%)	3031 (38%)	7068 (64%)	4050 (36%)
Age in 1992		44·7 (12·3)	50·2 (11·8)	44·7 (12·3)	49·9 (11·9)
Vital status at the end of 1998					
	Alive	4484 (89%)	2842 (94%)	6412 (91%)	3833 (95%)
	Deceased	534 (11%)	189 (6%)	656 (9%)	217 (5%)
Education					
	Elementary or incomplete secondary	1182 (24%)	906 (30%)	1546 (22%)	1302 (32%)
	Complete academic and vocational secondary	1926 (38%)	1032 (34%)	2952 (42%)	1290 (32%)
	Vocational higher education or incomplete higher	1367 (27%)	821 (27%)	1912 (28%)	1074 (27%)
	Complete academic higher education	543 (11%)	272 (9%)	658 (9%)	384 (10%)
Occupation in 1990s					
	Military	65 (1%)	4 (<1%)	100 (1%)	4 (<1%)
	Managerial	232 (5%)	77 (3%)	352 (5%)	98 (2%)
	High professional	587 (12%)	600 (20%)	647 (9%)	813 (20%)
	Low professional/routine non-manual	191 (4%)	485 (16%)	273 (4%)	675 (17%)
	Skilled manual	3039 (61%)	698 (23%)	4340 (61%)	765 (19%)
	Unskilled manual	268 (5%)	193 (6%)	409 (6%)	376 (9%)
	Unemployed in 1990s	636 (13%)	974 (32%)	947 (13%)	1319 (33%)
Marital status at the date of interview/death					
	Partnered	4464 (89%)	2554 (84%)	6416 (91%)	3447 (85%)
	Single	64 (1%)	91 (3%)	70 (1%)	105 (3%)
	Separated	490 (10%)	386 (13%)	582 (8%)	498 (12%)
Smoking status					
	Never smoked	1215 (24%)	2827 (93%)	1837 (26%)	3802 (94%)
	Currently/was a regular smoker	2879 (57%)	112 (4%)	3851 (55%)	142 (4%)
	Used to smoke but quit	924 (18%)	92 (3%)	1380 (20%)	106 (3%)
Alcohol consumption					
	Almost every day or several times a week	731 (15%)	23 (1%)	874 (12%)	35 (1%)
	About 2–4 times a month or up to once a month	2876 (57%)	1165 (38%)	3882 (55%)	1251 (31%)
	A couple of times a year	342 (7%)	427 (14%)	517 (7%)	561 (14%)
	Used to drink but quit	552 (11%)	67 (2%)	911 (13%)	80 (2%)
	Never	517 (10%)	1349 (45%)	884 (13%)	2213 (52%)
Material deprivation					
	Often or sometimes	119 (2%)	62 (2%)	202 (3%)	104 (3%)
	Rarely or never	4899 (98%)	2969 (98%)	6866 (97%)	3956 (97%)

Data are n (%) or mean (SD).

Fast privatisation was strongly associated with higher working-age male mortality rates both between 1992 and 1998 (age-standardised mortality ratio in men aged 20–69 years in fast *vs* slow privatised towns: 1·13, SMR 0·83 [95% CI 0·77–0·88] *vs* 0·73 [0·69–0·77], respectively) and from 1999 to 2006 (1·15, 0·91, 0·86–0·97 *vs* 0·79, 0·75–0·84). These preliminary results suggest that mortality rates were significantly higher in fast-privatised mono-industrial towns than in slow-privatised mono-industrial towns both in 1992–1998 and 1998–2006.

All towns except one in the sample initiated privatisation between 1992 and 1998, thus enabling us to test the privatisation hypothesis. Models 1 in table 2 and table 3 report age-adjusted incidence rate ratios, while models 2 and 3 include controls for sociodemographic and socioeconomic characteristics and health-related behaviours. As the towns were closely matched with respect to population size, dependency ratio, level of health provision, housing provision, pollution, initial mortality rate, alcohol poisoning, and level of income, these town-level variables were not included in the models. We controlled for individual-level education, occupation, material deprivation in the 1990s, marital status, and frequency of drinking and smoking.

**Table 2:** Age-adjusted incidence rate ratios of death from Poisson models in men aged 20–69 years between 1992 and 1998

		**Model 1**	**Model 2**	**Model 3**
Speed of privatisation (ref: slow speed)		1·15 (1·03–1·29)	1·17 (1·05–1·31)	1·13 (1·01–1·26)
Education (ref: elementary)				
	Complete academic and vocational secondary	..	0·99 (0·86–1·14)	1·04 (0·91–1·19)
	Vocational higher education or incomplete higher	..	0·83 (0·70–0·98)	0·93 (0·78–1·10)
	Complete academic higher education	..	0·70 (0·52–0·93)	0·82 (0·62–1·10)
Occupation (ref: unskilled manual)				
	Military	..	0·92 (0·47–1·79)	0·95 (0·49–1·97)
	Managerial	..	0·79 (0·43–1·19)	0·92 (0·62–1·38)
	High professional	..	0·73 (0·49–1·03)	0·80 (0·57–1·13)
	Low professional/routine non-manual	..	1·20 (0·73–1·75)	1·29 (0·88–1·88)
	Skilled manual	..	0·86 (0·60–1·09)	0·91 (0·72–1·16)
	Was not working in the 90s	..	1·32 (0·94–1·70)	1·40 (1·08–1·81)
Material deprivation (ref: rarely or never)				
	Often or sometimes	..	..	1·21 (0·91–1·61)
Marital status (ref: partnered)				
	Single	..	..	0·88 (0·48–1·63)
	Separated	..	..	1·19 (0·99–1·44)
Alcohol consumption (ref: a couple of times a year)				
	Almost every day or several times a week	..	..	1·58 (1·21–2·05)
	About 2–4 times a month or up to once a month	..	..	1·20 (0·94–1·53)
	Used to drink but quit	..	..	0·62 (0·45–0·85)
	Never	..	..	1·24 (0·93–1·67)
Smoking (ref: never smoked)				
	Used to smoke but quit	..	..	0·85 (0·69–1·05)
	Currently/was a regular smoker	..	..	1·67 (1·44–1·95)

Data are incidence rate ratio (95% CI). Data are for 12 086 men and 1190 events. ref=reference.

**Table 3:** Age-adjusted incidence rate ratios of death from Poisson models among female cohorts aged 20–69 between 1992 and 1998

		**Model 1**	**Model 2**	**Model 3**
Speed of privatisation ref: slow speed)		1·16 (0·95–1·40)	1·17 (0·97–1·42)	1·18 (0·97–1·43)
Education (ref: elementary)				
	Complete academic and vocational secondary	..	0·93 (0·73–1·19)	0·93 (0·73–1·19)
	Vocational higher education or incomplete higher	..	1·08 (0·80–1·45)	1·08 (0·80–1·45)
	Complete academic higher education	..	0·64 (0·37–1·10)	0·64 (0·37–1·10)
Occupation (ref: unskilled manual)				
	Military	..	0·00 (0·00–0·00)	0·00 (0·00–0·00)
	Managerial	..	1·09 (0·44–2·72)	1·11 (0·45–2·78)
	High professional	..	0·88 (0·50–1·54)	0·89 (0·51–1·56)
	Low professional/routine non-manual	..	1·04 (0·63–1·73)	1·04 (0·63–1·73)
	Skilled manual	..	0·70 (0·42–1·17)	0·71 (0·42–1·18)
	Was not working in the 90s	..	1·56 (1·02–2·37)	1·57 (1·04–1·39)
Material deprivation (ref: rarely or never)				
	Often or sometimes	..	..	1·35 (0·78–2·33)
Marital status (ref: partnered)				
	Single	..	..	1·18 (0·68–2·05)
	Separated	..	..	1·03 (0·73–1·45)
Alcohol consumption (ref: a couple of times a year)				
	Almost every day or several times a week	..	..	1·78 (0·59–5·35)
	About 2–4 times a month or up to once a month	..	..	0·90 (0·66–1·23)
	Used to drink but quit	..	..	0·70 (0·31–1·60)
	Never	..	..	0·91 (0·69–1·21)
Smoking (ref: never smoked)				
	Used to smoke but quit	..	..	1·11 (0·57–2·19)
	Currently/was a regular smoker	..	..	0·99 (0·47–2·11)

Data are incidence rate ratio (95% CI). Data are for 7081 women and 406 events. ref=reference.

The incidence rate ratio for fast privatisation in the basic (age-adjusted) model 1 was 1·15 (95% CI 1·03–1·29) times higher than for the slow group for male relatives (table 2). In model 2 (main model), with controls for education and occupation, the incidence rate ratio was 1·17 (95% CI 1·05–1·31) times higher for men. In the fully adjusted models 3, which additionally include variables measuring material deprivation, alcohol consumption, smoking and marital status, the association with privatisation is attenuated but remains important with mortality 1·13 times higher (95% CI 1·01–1·26) in fast-privatised settlements, a similar estimate to that obtained in the comparison of SMRs. The model-predicted estimates of the mortality rates in fast privatisation and slow privatisation towns are 0·014 and 0·016, respectively. All control variables showed effects that were in the expected directions. We re-fitted the models 1–3 for both sexes on a dataset with imputed missing values using multivariable imputation via chained equations and the resulting coefficients were in line with those presented in table 4.

**Table 4:** Age-adjusted incidence rate ratios of death from Poisson models (robustness check)

	**Incidence rate ratio**	**N**	**N (events)**
**Men**			
1992–1998, main model	1·17 (1·05–1·31)	12 086	1190
1992–1998, main model, hierarchical	1·17 (1·01–1·36)	12 086	1190
1992–1998, main model, for older cohorts*	1·15 (1·03–1·30)	7768	1061
5 years after privatisation, main model	1·21 (1·08–1·37)	11 819	1062
1992–2006, main model	1·16 (1·08–1·23)	12 086	3288
1999–2006, main model	1·18 (1·08–1·30)	10 315	1714
**Women**			
1992–1998, main model	1·17 (0·97–1·42)	7081	406
1992–1998, main model, hierarchical	1·20 (0·92–1·56)	7081	406
1992–1998, main model, for older cohorts*	1·18 (0·97–1·43)	5709	394
Five years after privatisation, main model	1·24 (1·01–1·51)	6928	382
1992–2006, main model	1·14 (1·03–1·26)	7081	1431
1999–2006, main model	1·13 (0·97–1·32)	5794	623

Data are incidence rate ratio (95% CI) or n. The coefficients give the hazard ratio of death in relation to an absolute change in the independent variable (privatisation speed). Relative hazard ratios are presented with 95% CIs based on robust (heteroscedasticity-corrected) standard errors. We report age-adjusted hazard ratios of death from Poisson models among cohorts aged 20–69 years, with covariates: individuals' occupation, educational level.

* Aged 40–69 years.

The findings in men changed little when we fitted our model with different, more conservative specifications. While the short-to-medium term effect of the rapid privatisation was captured in the main model, we explored long-term effect of privatisation on health and mortality in models based on the data for longer (1992–2006) and later (1999–2006) periods. In these models the effect of privatisation was significant in all specifications for men, and significant for women in a model covering the longer period (table 4). The magnitude of the privatisation effect was slightly larger than for 1992 to 1998 and significance levels were not changed. When we restricted the sample to cohorts of 40 years and older, point estimates and significance levels were unaffected (table 4).

We also fitted a Poisson model in which we took into account the actual year of privatisation. Although most towns experienced privatisation in 1993, some privatisation started in 1992. In this model we specified the start of exposure as the year of privatisation, and the end of exposure 5 years after the start of privatisation (table 4). Taking into account individuals' occupation and educational level, the incidence rate ratio of living in a town with rapid privatisation in this specification for men was 1·21 (95% CI 1·08–1·37).

The findings were also robust when we used multi-level regressions with town-level random effects. Incidence rate ratios for males from this model (1·17, 95% CI 1·01–1·36) were very similar to the findings of the default models for men (table 4). The next two models analysed the mortality during the pre-intervention period: from 1985 to 1991. In these placebo analysis models (appendix p 10) mortality differentials between towns where the city-forming enterprise underwent slow or fast privatisation disappeared. As a final placebo analysis, we compared the relatives from fast and gradual privatised towns who did not live in those towns for a large part of the 1990s. There was no difference in the mortality rate between those groups (appendix p 10).

Results for women showed weaker effects, with sizes varying from 13 to 24 in most models but rarely reaching significance. Only in the model accounting for the actual year of privatisation was the female incidence rate ratio large and significant (1·24, 95% CI 1·01–1·51; table 4).

## Discussion

The PrivMort project, to our knowledge, is the largest multi-level indirect retrospective cohort study to date done in the post-communist countries. We found clear differences in mortality in working-age men between towns where the city-forming enterprise underwent slow rather than fast privatisation. Working-age men in fast-privatised towns experienced 13% to 21% higher mortality than working-age men in slow-privatised towns, depending on the specification or sample restrictions applied. Findings in women were broadly consistent but not significant, mainly due to the smaller number of deaths in women.

Our results are consistent with a previous cross-national study by Stuckler and colleagues,^10^ which noted that rapid privatisation was associated with a rise in male death rates. The magnitude of association in their findings,^10^ a 12·8% (95% CI 7·9–17·7) rise linked with introduction of mass privatisation was lower than in the present study (17%, 95% CI 5–31). This finding is unsurprising in view of the different operationalisation of privatisation used in the study by Stuckler and colleagues.^10^ The greater estimate in this study is to be expected because the national-level finding estimates mortality in all towns and cities, both gradual and fast privatisers. Additionally, we were better able to isolate the effect of privatisation. Whereas in the previous study^10^ the percentage change in mortality ratio was adjusted for the European Bank for Reconstruction and Development (EBRD) price liberalisation index, EBRD trade liberalisation index, the democratisation index, dummy for military or ethnic conflict, the percentage of urban population, dependency ratio, per capita log gross domestic product, and education level, here we adjusted for all of these characteristics and a wider set of variables. The first five covariates were accounted for by restricting the analysis to the urban population of a single country. The next two covariates were accounted for by matching towns on dependency ratio and average wage; additionally, we matched on other socioeconomic and demographic variables. Unlike the previous work,^10^ we were able to adjust for education on the level of individuals, not country level, which makes our estimates more precise.

Previous researchers have suggested that women have been able to cope with the post-Soviet transitions better than men.^29^ Although Russian women faced a double burden from participating in the labour market and taking care of their families,^30^, ^31^ paradoxically this greater responsibility seems to have given their lives more meaning. It has also been suggested that, faced with unemployment, Russian men turned to cheap and easily available alcohol to seek compensation for their lost provider's status,^32^ while heavy alcohol consumption was stigmatised among women.^33^

The indirect estimation technique we used adequately assesses overall mortality levels and differences in mortality between different socioeconomic subgroups, as findings of several large-scale studies in Russia have previously shown.^16^, ^34^, ^35^, ^36^ In fact, the present study has several advantages common to indirect techniques of estimating mortality, mainly in terms of cost and time efficiency and ability to retrieve information about people usually unreachable by direct techniques. Additionally, the PrivMort study retrieved a broad range of socioeconomic and demographic information not otherwise available.

However, our study has limitations. First, the sample was not representative of the entire population of Russia because we focused only on the urban population of industrial mono-enterprise towns in the European part of the country. Moreover, the fact that national statistics average across the whole population of Russia—including the Muslim population with lower reported alcohol consumption^37^—makes it difficult to compare these figures with the sample of 20 towns that have a mainly Russian population. The total number of Muslims is estimated to be lower than 2·8 million, or less than 2% of Russia's total population.^38^ Second, potential selection bias might have arisen from differentials in family-wise mortality levels: representatives from high-mortality families were less likely to have participated in the survey because a higher proportion would not survive until the interview date, making such families less likely to be captured by the sampling, and pass the screening criteria for the interviews, which require respondents' relatives to reside in the settlements in question. However, research by Murphy and colleagues^27^ suggests that this is not likely to bias the mortality estimates. Third, as those who emigrated away from the 20 settlements were excluded from the study, future investigations should look into the migrant differentials at settlement level. Possible bias might arise from out-migration from fast privatisation towns; however, this could both overestimate and underestimate the effect of the speed of privatisation. If healthy people left a fast privatisation town, this could introduce an upward bias, if families who lost their fathers (ie, the main earners) left the town, this would contribute to underestimation of the effect of privatisation on mortality.

The fourth concern relates to temporal inconsistency of the recorded individual characteristics and mortality outcomes. While individual characteristics such as education, marital status, alcohol consumption, and smoking were recoded at the time of death for decedents (between 1992 and 1998), for surviving relatives these characteristics were collected at time of the interview and at the time of death for those who died after 1998, which might be as late as 2015. However, because we were only studying relatives aged 20 years and older, and, in view of low transition rates to higher education for adults in Russia, education status is unlikely to change. Marital status and alcohol and tobacco use might have changed between the exposure period and the date of interview, hence the result of the full models 3 must be treated with caution. Finally, there were potential problems in accurately recalling events that happened 20–25 years ago; this can potentially bias the estimates of frequency of drinking and smoking, education, and frequency of communication for relatives. Anticipating this, interviewers were instructed to introduce auxiliary sentences and memory cards to facilitate accurate recall and obtain accurate estimates of characteristics and behaviours of their relatives. However, for all five of these biases, there was no reason to think they would vary with the speed of privatisation in the settlements.

The method of isolating the effect of privatisation by closely matching the settlements before collecting the data also entails certain limitations. We deployed propensity score matching on baseline covariates that are most important to health outcomes, but one could argue that fast-privatised towns are systematically different from slow-privatised towns in some other ways. A systematic literature review of the causes of privatisation^15^ showed that the main determinants of privatisation were political, not underlying economic weakness or social conditions. The political choice of the speed of privatisation was made on the level of enterprises or local and regional authorities, thus it can be claimed that it was probably orthogonal to mortality outcomes.

Clearly, there are several factors contributing to excess mortality in Russia, acting at different points along several causal pathways, with alcohol playing a key role in several, including that related to speed of privatisation. In a study by Walberg and colleagues^5^ that reported an association between labour turnover in Russian regions, itself linked with privatisation, researchers noted that much of the excess mortality was alcohol-related. Treisman^39^ invoked the fall in alcohol prices from 1990 to 1994 as the primary determinant of increased mortality during the 1990s. Treisman's measure of price was based on the cost of vodka in the capital city of each of Russia's regions. There were no data available for alcohol prices at the settlement level that would allow us to directly test this hypothesis. Another possible component of several causal pathways involves social protection. Increased mortality could be related to variations in the provision of social benefits at the settlement level. However, local expenditures are strongly dependent on the health of city-forming enterprises.^15^ Similarly, mortality could be related to sector or firm-specific characteristics, possibly operating through lower wages. However, without data for these variables, we cannot test all possible causal pathways.

It is essential to recognise that the health effects of transition were complex and defy mono-causal explanation. We do not claim that mass privatisation was the only cause of increased mortality. In fact, it would be remarkable if one single policy or factor explained all the variation in the post-communist mortality crisis. We believe our findings provide strong evidence for the hypothesis that rapid privatisation contributed to raised working-age male mortality. Importantly, the findings of this study complement the epidemiological and public health explanations linking alcohol consumption and psychological stress to the Russian mortality crisis of 1992 to 1998,^40^ identifying rapid privatisation as an important underlying cause. Regression coefficients for fast privatisation were consistently positive and significant for men, but usually not significant for women. Robustness checks did not significantly change the magnitude or the direction of the covariates of mortality. This study adds to previous, less conclusive findings on the effects of economic governance on all-cause mortality risk in working-age men, and makes a valuable methodological and empirical contribution to the topic.
