# Supplementary material for: The effect of rapid privatisation on mortality in mono-industrial towns in post-Soviet Russia: a retrospective cohort study
Source: Lancet Public Health. 2017 Apr 11;2(5):e231–8. doi: 10.1016/S2468-2667(17)30072-5 (PMC5459934; doi:10.1016/S2468-2667(17)30072-5)
Supplement: Supplementary appendix [file mmc1.pdf]

THE LANCET
Public Health

Supplementary appendix

This appendix formed part of the original submission and has been peer reviewed. We post it as supplied by the authors.

Supplement to: Azarova A, Irdam D, Gugushvili A, et al. The effect of rapid privatisation on mortality in mono-industrial towns in post-Soviet Russia: a retrospective cohort study. *Lancet Public Health* 2017; published online April 11. http://dx.doi.org/10.1016/S2468-2667(17)30072-5.

Supplementary Web Appendix

This web appendix forms a part of the original submission and has been peer-reviewed.

Supplement to Aytalina Azarova; Darja Irdam; Alexi Gugushvili; Mihaly Fazekas; Gábor Scheiring; Pia Horvat; Denes Stefler; Irina Kolesnikova; Vladimir Popov; Ivan Szelenyi; David Stuckler, Michael Marmot; Michael Murphy; Martin McKee; Martin Bobak; Lawrence King **The effect of rapid privatisation on mortality in mono-industrial towns in post-Soviet Russia: a retrospective cohort study.**

Web Appendix 1. List of study towns

Web Appendix 2. Means of covariates, used in propensity score matching, in two groups of towns in Russia

Web Appendix 3. Replication of the main model, hierarchical specification, exposure years: 1992-1998

Web Appendix 4. Replication of the main model on older cohorts, exposure years: 1992-1998

Web Appendix 5. Replication of the main model with actual privatization year as an onset of exposure

Web Appendix 6. Replication of the main model, exposure years: 1992-2006

Web Appendix 7. Replication of the main model, exposure years: 1999-2006

Web Appendix 8. Additional sensitivity analysis: models on non-residents, exposure years 1992-1998 and on residents, exposure years: 1985-1991

Web Appendix 9. Description of study variables

Web Appendix 10. Description of Privmort survey design

Web Appendix 1

List of study towns

Town	Sub-national unit
Sel’tso	Bryanskaya oblast
Nikol’sk	Penzenskaya oblast
Belinsky	Penzenskaya oblast
Plavsk	Tulskaya oblast
Bakal	Chelyabinskaya oblast
Sim	Chelyabinskaya oblast
Danilov	Yaroslavskaya oblast
Starodub	Bryanskaya oblast
Boguchar	Voronezhskaya oblast
Dalmatovo	Kurganskaya oblast
Karabash	Chelyabinskaya oblast
Mtsensk	Orlovskaya oblast
Yasnogorsk	Tulskaya oblast
Zhukovka	Bryanskaya oblast
Nieman	Kaliningradskaya oblast
Navoloki	Ivanovskaya oblast
Lakinsk	Vladimirskaya oblast
Kohma	Ivanovskaya oblast
Nikolsk	Vologdskaya oblast
Privolzhsk	Ivanovskaya oblast

Web Appendix 2

Means of covariates, used in propensity score matching, in two groups of towns in Russia

Covariates	Rapidly privatised towns		Gradually privatised towns	
	mean	sd	Mean	sd
Number of deaths per 1000 population	12·7	2·7	13·3	3·24
Population, persons	21,010	11,387	17,980	5,808
Old-age dependency ratio	0·83	0·09	0·84	0·09
Average wages in US dollars, per month	16·5	2·6	14·7	5·4
Alcohol poisoning per 100,000 population	16·5	2·6	17·1	6·3
Number of physicians per 10,000 population	27·0	9·0	30·6	12·5
Floor area per person, square meters	17·7	7·6	17·1	6·3
Emission of pollutants into atmosphere, 1000 tons	0·76	1·7	0·28	2·1

Web Appendix 3

We replicated the main model on hierarchical Poisson specification to account for intra-settlement heterogeneity.

	Males (N = 12,086, N of events =1,190)			Females (N=7,081, N of events =406)		
	IRR	[95% Conf·	Interval]	IRR	[95% Conf·	Interval]
**Speed of privatization (ref: slow**	1·17	1·01	1·36	1·2	0·92	1·56
Education (ref: elementary)						
Complete academic and vocational secondary	1	0·87	1·15	0·92	0·72	1·18
Vocational higher education or incomplete higher	0·84	0·7	0·99	1·08	0·81	1·44
Complete academic higher education	0·71	0·53	0·95	0·65	0·38	1·11
Occupation in 90s (ref: unskilled manual)						
Military	0·92	0·47	1·8	0	0	0
Managerial	0·79	0·52	1·19	1·1	0·44	2·74
High professional	0·73	0·51	1·04	0·87	0·51	1·5
Low professional/routine non-manual	1·21	0·82	1·79	1·04	0·62	1·72
Skilled manual	0·86	0·67	1·1	0·7	0·41	1·17
Was not working in 90s	1·31	1	1·7	1·56	1·02	2·4
Constant	0	0	0	0	0	0
Random effects:						
Settlement-level variance	0·106	0·049	0·231	0·191	0·091	0·399

Web Appendix 4

We replicated the model on the same exposure period as a main model, 1992-1998, but on older cohorts: 40-69 years old at the onset of exposure.

	Males (N = 7,768, N of events = 1,061)			Females (N=5,709, N of events=394)		
	IRR	[95% Conf· Interval]		IRR	[95% Conf· Interval]	
Speed of privatization (ref: slow speed)	1·15	1·03	1·30	1·18	0·97	1·43
Education (ref: elementary)						
Complete academic and vocational secondary	0·96	0·84	1·11	0·93	0·73	1·19
Vocational higher education or incomplete higher	0·85	0·71	1·01	1·10	0·82	1·48
Complete academic higher education	0·76	0·57	1·02	0·60	0·34	1·07
Occupation (ref: unskilled manual)						
Military	0·43	0·16	1·18	0·00	0·00	0·00
Managerial	0·73	0·48	1·11	0·95	0·36	2·55
High professional	0·68	0·47	0·97	0·89	0·51	1·56
Low professional/routine non-manual	1·13	0·75	1·71	0·98	0·58	1·65
Skilled manual	0·80	0·62	1·03	0·62	0·37	1·07
Was not working in 90s	1·22	0·94	1·59	1·50	0·99	2·29
Constant	0·01	0·01	0·02	0·00	0·00	0·01

Web Appendix 5

We replicated the main model with actual privatization year as an onset of exposure

	Males (N = 11,819, N of events = 1,062)			Females (N=6,928, N of events=382)		
	IRR	[95% Conf· Interval]		IRR	[95% Conf· Interval]	
Speed of privatization (ref: slow	1·21	1·08	1·37	1·24	1·01	1·51
Education (ref: elementary)						
Complete academic and vocational secondary	0·97	0·83	1·12	0·95	0·74	1·22
Vocational higher education or incomplete higher	0·85	0·71	1·02	0·95	0·69	1·30
Complete academic higher education	0·67	0·49	0·92	0·60	0·34	1·06
Occupation (ref: unskilled manual)						
Military	1·27	0·68	2·36	0·00	0·00	0·00
Managerial	0·88	0·58	1·35	1·12	0·45	2·77
High professional	0·79	0·55	1·15	0·97	0·56	1·68
Low professional/routine non-manual	1·14	0·75	1·73	0·87	0·52	1·44
Skilled manual	0·92	0·70	1·19	0·61	0·37	1·02
Was not working in 90s	1·38	1·05	1·82	1·42	0·95	2·13
Constant	0·00	0·00	0·00	0·00	0·00	0·00

Web Appendix 6

We replicated the main model for a longer period: 1992-2006.

	Males (N = 12,086, N of events = 3,288)			Females (N=7,081, N of events=1,431)		
	IRR	[95% Conf· Interval]		IRR	[95% Conf· Interval]	
Speed of privatization (ref: slow speed)	1·16	1·08	1·23	1·14	1·03	1·26
Education (ref: elementary)						
Complete academic and vocational secondary	0·90	0·83	0·97	0·99	0·88	1·12
Vocational higher education or incomplete higher	0·73	0·66	0·80	1·06	0·91	1·22
Complete academic higher education	0·59	0·50	0·70	0·77	0·60	1·00
Occupation (ref: unskilled manual)						
Military	0·77	0·52	1·13	0·00	0·00	0·00
Managerial	0·59	0·46	0·76	1·00	0·64	1·58
High professional	0·77	0·64	0·92	0·88	0·68	1·14
Low professional/routine non-manual	0·75	0·59	0·96	0·83	0·64	1·07
Skilled manual	0·81	0·71	0·93	0·93	0·74	1·18
Was not working in 90s	1·12	0·97	1·29	1·28	1·04	1·58
Constant	0·01	0·00	0·01	0·00	0·00	0·00

Web Appendix 7

We replicated the main model for a later period: 1999-2006.

	Males (N = 10,315, N of events = 1,714)			Females (N=5794, N of events=623)		
	IRR	[95% Conf· Interval]		IRR	[95% Conf· Interval]	
Speed of privatization (ref: slow	1·18	1·08	1·30	1·13	0·97	1·32
Education (ref: elementary)						
Complete academic and vocational secondary	0·83	0·74	0·93	0·87	0·71	1·06
Vocational higher education or incomplete higher	0·65	0·57	0·75	0·86	0·68	1·09
Complete academic higher education	0·53	0·41	0·67	0·65	0·44	0·96
Occupation (ref: unskilled manual)						
Military	0·68	0·41	1·12	0·00	0·00	0·00
Managerial	0·44	0·31	0·63	1·09	0·59	2·00
High professional	0·73	0·57	0·93	1·01	0·70	1·46
Low professional/routine non-manual	0·51	0·36	0·72	0·83	0·58	1·20
Skilled manual	0·75	0·63	0·90	1·11	0·80	1·54
Was not working in 90s	0·98	0·79	1·22	1·45	1·08	1·96
Constant	0·00	0·00	0·01	0·00	0·00	0·01

Web Appendix 8

We replicated the models on a population of non-residents for a period 1992-1998 and on a population of residents for pre-transition period: 1999-2006.

	Population: non-residents, exposure 1992-1998						Population: residents, exposure 1985-1991					
	Males (N = 2,419 N of events=296)			Females (N=2,438, N of events=114)			Males (N = 11,539, N of events=148)			Females (N=7,052, N of events=49)		
	IRR	[95% Conf.	Interval]	IRR	[95% Conf.	Interval]	IRR	[95% Conf.	Interval]	IRR	[95% Conf.	Interval]
Speed of privatization (ref: slow speed)	0·72	0·51	1·02	1·48	1·03	2·13	0·72	0·51	1·02	0·82	0·46	1·45
Constant	0·00	0·00	0·00	0·00	0·00	0·01	0·00	0·00	0·00	0·00	0·00	0·00

Web Appendix 9

Description of study variables

Outcome variable: coded 0 if a relative survived beyond the end of 1998, i.e. was alive at the date of an interview or died in 1999 or later.

Privatization speed: coded 0 if less than 50% of state shares of the city-forming enterprise were privatized in any two consecutive years between 1992 and 1998, and 1 if 90% or more of city-forming enterprise's shares were privatized in any of two consecutive years between 1992 and 1998.

Education was defined at the highest level completed as an answer to the following question:

What is the highest level of education your father/your mother/your sibling/your partner (has) achieved?

1. Incomplete elementary education
2. Complete elementary education or incomplete secondary
3. Complete academic secondary education (graduated from high school, lyceum, gymnasium)
4. Complete vocational secondary education without general high school leaving exam (elementary vocational education or vocational training which do not give access to higher education)
5. Complete vocational secondary education with general high school leaving exam (PTU, SPTU which give access to higher education)
6. Incomplete higher education
7. Complete vocational higher education (graduated from technical school, military, pedagogical, medical college – except those which give full academic higher education degrees)
8. Complete academic higher education (graduated from at least one institute of higher education – institute, university, academy)

The relatives were grouped into four levels of education: "Elementary" (answers numbered 1 and 2); "Complete academic and vocational secondary" (answers numbered 3, 4 and 5); "Vocational higher education or incomplete higher" (answers numbered 6 and 7); and "Complete academic higher education" (answer number 8).

Position: This variable was coded “Military” if a respondent indicated that his relative’s occupation was in military forces or police; “Managerial” if relative’s occupation was one of the following: public official, lawmaker director/chief executive, head of department, or head of company; “High Professional” if relative’s occupation was one of the following: technical specialist and computer engineer specialist, lecturer or teacher (including methodologist), finance and accounting specialist, life science specialist, healthcare specialist, social science and art specialist, librarian, religious official, technical specialists, such as in physical and machine-building, optical equipment operator or agronomy and forestry technician, assistant in any area, security and quality control agent, detective, officer of justice, marine vessel and aircraft specialist, art specialist, or office worker who deals with office work or finance; “Low professional/routine non-manual” if relative’s occupation was one of the following: office worker in accountancy/administrative office/post office, worker engaged in typing, post and transport employee, other clerk, trade and services employee, employee engaged in processing , service workers and security workers; “Skilled manual” if relative’s occupation was one of the following: ‘market’ gardener, farmer agriculture workers, animal breeder, fishing worker, mining and construction worker, worker engaged in metallurgy/machine-building and related industries, worker engaged in precise measurements, craftsman, equipment operator, machine minder, or machinery operator; “Unskilled manual” if relative’s occupation was one of the following: street vendor, shoe shiner, home helper or cleaner, courier, dustman, non-qualified personnel employed in agriculture, construction or mining, or other non-qualified manual job.

Material deprivation was defined as dummy variable based on the answer to the following question: Did your father/your mother/your sibling/your partner ever have to go without things people really need, like food, heat or clothes (i.e., suffered material hardship) for long periods in the 1990s? Answers “Never” or “Rarely” were coded as “Rarely or never” and answers “Sometimes” and “Often” were coded as “Often or sometimes”.

Marital status was coded “Partnered” if relative’s marital status was indicated by respondent to be “Married or living with long-term partner” or “Widowed”; “Single” if indicated as “Single”; “Separated” if indicated as “Divorced” or “Still married, but separated”. Husbands who were not divorced were coded as “Partnered”, and divorced husbands were coded as re-married and therefore “Partnered”.

The relatives were grouped into three levels of **smoking status,** depending on the answer to the following question:

Does/did your father/your mother/your sibling/your partner smoke? If yes, how much?

1. Never smoked
2. Used to smoke but quit
3. Regular smoker

Relative was coded as “Never smoked” if the answer was “Never smoked”, coded as “Used to smoke but quit”, if the answer was “Used to smoke but quit”, and coded as “Currently/was a regular smoker” if the answer was “Regular smoker”.

The variable for **alcohol consumption** was coded on the basis of the answer to the following question:

Does/did your father/your mother/your sibling/your partner drink alcoholic beverages? If yes, how often?

1. Almost every day
2. Several times a week
3. About 2-4 times a month
4. Up to once a month (on special occasions)
5. A couple of times a year
6. Used to drink, but quit
7. Never drank

The relatives were grouped into five levels of alcohol consumption: “Almost every day or several times a week” (answers numbered 1 and 2); “About 2-4 times a month or up to once a month” (answers numbered 3 and 4); “A couple of times a year” (answer number 5); “Used to drink but quit” (answer number 6); and “Never” (answer number 7).

Web Appendix 10

Description of Privmort survey design

In the PrivMort project, multi-level retrospective convenience cohort study, interviews with randomly selected respondents collected information on their closest relatives. The survey was conducted by the Russian Agency for Public Opinion Research (VCIOM) between November 2014 and March 2015. Data were collected in face-to-face PAPI interviews using structured questionnaires, covering socio-demographic characteristics of relatives, including the residency history (including some questions on international and domestic migration); vital status; education levels; marital status; smoking and alcohol consumption; labour market position and employment history; and history of material deprivation. In total, there were 14,642 collected valid interviews, with a 65% overall response rate, 68% in fast and 63 in slow privatized towns.

We used a 'random walk' procedure for sampling the households. First, a map of each settlements was divided into numbered cells, and the starting points for 'random walk' routs were identified by generating random numbers. Second, an interviewer started a route from a primary sampling unit located at the centre of the selected cell ensuring that at least three eligible households are between the household where interview took place and the next household. He or she continued the 'walk' until 25 interviews were conducted.

At each randomly selected primary sampling unit only one respondent was selected, even in cases when more than one family shared the same house. In cases where more than one person in the household matched the screening criteria (older than 42 years of age; relatives lived in the same settlement between 1980 and 2010), the person whose birthday is closer to the date of the survey was selected for the interview. Interviewers had to make four attempts at interviewing the person who matched the screening criteria if he or she was temporarily unavailable.

All respondents were born before 1972 to ensure that they and their relatives were of working age in 1991 and hence could potentially be affected by the transition. The selection was conditional on the fact that their family members lived in the same settlement for a prolonged period of time during and after the transitions. While the criterion for women was to have at least one family member (parents, siblings or a spouse) living in the same settlement in this time period, the survey excluded male respondents who only had their spouses residing in the same settlement.

The convenience cohort consists of three types of relatives of the respondents in the population surveys: parents, siblings and spouses/partners. Information on survival is collected for a maximum of two (oldest) siblings who survived to age 20, the age at which our analysis of adult mortality starts. The third group of relatives consists of the first partners (married or long-term cohabiters) of female respondents. We ask about survival of the first partner, since if current partner were included, information on men who died early would be differentially excluded and therefore bias results.

The questionnaire was developed by a multidisciplinary team of researchers, followed by cognitive testing on respondents sampled from mono-industrial towns using the snowball method. The cognitive tests were carried out in a controlled environment to identify problematic wording and sensitive questions. As a result of the cognitive interviews, the questionnaire has been modified to ensure a smooth flow and to make the respondents as comfortable and confident as possible. During the cognitive tests we discovered that respondent sensitivity was less of a problem than initially feared, consistent with previous experience in Russia where people often appreciated the opportunity to talk about their deceased relatives.

VCIOM has performed back-checks for at least 10 per cent of complete interviews conducted in each settlement and 15 per cent of unsuccessful interview attempts. The back-checks were mostly performed by phone, while in some cases the regional supervisors carried out the back-checks by visiting individual households.

The survey has been approved by the University of Cambridge Department of Sociology ethics committee. The data were anonymized to prevent any potential identification of individual respondents.
